# The level of countries’ preparedness to health risks during Covid-19 and pre-pandemic: the differential response to health systems building blocks and socioeconomic indicators

**DOI:** 10.1186/s13561-023-00428-9

**Published:** 2023-03-14

**Authors:** Omar B. Da’ar, Farah Kalmey

**Affiliations:** 1 Department of Health Systems Management, College of Public Health and Health Informatics, King Saud bin Abdulaziz University for Health Sciences, Riyadh, Saudi Arabia; 2 King Abdullah International Medical Research Center, Riyadh, Saudi Arabia; 3 Institute for Cost Analysis and Research Evaluation, Minneapolis, MN USA; 4 College of Science and Health Professions, King Saud bin Abdulaziz University for Health Sciences, Riyadh, Saudi Arabia; 5 Organizational Health and Wellbeing at the Division of Health Research, Lancaster University, Lancaster, UK

**Keywords:** Global health security, Health systems building blocks, Pandemic preparedness, And quantile regression, I100, I140, I150, I180

## Abstract

The global health security (GHS) Index assesses countries’ level of preparedness to health risks. However, there is no evidence on how and whether the effects of health systems building blocks and socioeconomic indicators on the level of preparedness differ for low and high prepared countries. The aim of this study was to examine the contributions of health systems building blocks and socioeconomic indicators to show differences in the level of preparedness to health risks. The study also aimed to examine trends in the level of preparedness and the World Health Organization (WHO) regional differences before and during the Covid-19 pandemic. We used the 2021 GHS index report data and employed quantile regression, log-linear, double-logarithmic, and time-fixed effects models. As robustness checks, these functional form specifications corroborated with one another, and interval validity tests confirmed. The results show that increases in effective governance, supply chain capacity in terms of medicines and technologies, and health financing had positive effects on countries’ level of preparedness to health risks. These effects were considerably larger for countries with higher levels of preparedness to health risks. The positive gradient trends signaled a sense of capacity on the part of countries with higher global health security. However, the health workforce including doctors, and health services including hospital beds, were not statistically significant in explaining variations in countries’ level of preparedness. While economic factors had positive effects on the level of preparedness to health risks, their impacts across the distribution of countries’ level of preparedness to health risks were mixed. The effects of Social Development Goals (SDGs) were greater for countries with higher levels of preparedness to health risks. The effect of the Human Development Index (HDI) was greatest for countries whose overall GHS index lies at the midpoint of the distribution of countries’ level of preparedness. High-income levels were associated with a negative effect on the level of preparedness, especially if countries were in the lower quantiles across the distributions of preparedness. Relative to poor countries, middle- and high-income groups had lower levels of preparedness to health risks, an indication of a sense of complacency. We find the pandemic period (year 2021) was associated with a decrease in the level of preparedness to health risks in comparison to the pre-pandemic period. There were significant WHO regional differences. Apart from the Eastern Mediterranean, the rest of the regions were more prepared to health risks compared to Africa. There was a negative trend in the level of preparedness to health risks from 2019 to 2021 although regional differences in changes over time were not statistically significant. In conclusion, attempts to strengthen countries’ level of preparedness to health shocks should be more focused on enhancing essentials such as supply chain capacity in terms of medicines and technologies; health financing, and communication infrastructure. Countries should also strengthen their already existing health workforce and health services. Together, strengthening these health systems essentials will be beneficial to less prepared countries where their impact we find to be weaker. Similarly, boosting SDGs, particularly health-related sub-scales, will be helpful to less prepared countries. Moreover, there is a need to curb complacency in preparedness to health risks during pandemics by high-income countries. The negative trend in the level of preparedness to health risks would suggest that there is a need for better preparedness during pandemics by conflating national health with global health risks. This will ensure the imperative of having a synergistic response to global health risks, which is understood by and communicated to all countries and regions.

## Background

This paper examines the relationship between global health security (GHS) versus health system building blocks, socioeconomic indicators, and the World Health Organization (WHO) regional differentials. The overall global health risk preparedness index is constructed as a composite measure, which is a linear combination of the pillars of GHS, including prevention, detection, rapid response, robust health system, compliance with international norms, and overall risk environment and vulnerability [1]. Specifically, we consider the questions: (a) What are the levels of contributions of a country’s health system building blocks and socioeconomic indicators to the level of preparedness to epidemics and pandemics? (b) Do the effects of the health systems building blocks and socioeconomic indicators differ for countries with weak and strong levels of preparedness to epidemics and pandemics? (c) What is WHO regional differentials in GHS during Covid-19 and pre-pandemic periods?

WHO defines global health security as the prevention, detection, and response to naturally emerging, accidental, and deliberate biological threats [2]. The Center for Disease Control (CDC) considers GHS as the existence of a strong and resilient public health system that can prevent, detect, and respond to infectious disease threats wherever they occur in the world [3].

The motivation for this research is that with increasing epidemics and pandemics globally, the level of countries’ health risk preparedness is now being measured to provide a framework to assess the capacity of countries to prevent and mitigate emerging health risks [1, 4]. The GHS Index was released prior to the Covid-19 pandemic in 2019. Recent work by the John Hopkins Center for Health Security 2021 Global Health Security Report provides a new composite measure of the level of preparedness to epidemics and pandemic threats for 195 countries [1]. The GHS Index also allows for the benchmarking of the overall score of a country’s level of preparedness against socioeconomic factors. These factors include income level, the Human Development Index (HDI), and Sustainable Development Goals (SDGs) [1]. The value preposition of improvements in socioeconomic indicators in protecting population health cannot be denied. The SDGs, for instance, emphasize strengthening of early warning, risk reduction, and management of health risks by the year 2030 [5]. HDI remains a valuable tool for guiding decision making and monitoring policymaking at both national and subnational levels, especially on health security capacities and capabilities in many countries [6].

The emergence and spread of infectious diseases with pandemic potential occurred regularly throughout history. Although public health control efforts have been in place for more than a century [7], protecting the health and safety of people remains an imperative for governments [8]. Despite improvements in technologies, communication, and health systems, countries around the world still face a perfect storm of converging threats that might substantially increase the risk of infectious disease epidemics [8]. In particular, the last two decades saw the emergence and reemergence of more deadly outbreaks, epidemics, and pandemics of infectious diseases causing widespread disruptions to all aspects of *global health* systems. Some of the diseases the world witnessed in recent decades include severe acute respiratory syndrome (SARS) coronavirus outbreak [9], H1N1 influenza [10], cholera [11], Middle East respiratory syndrome coronavirus (MERSCoV) [12], Ebola [13], Chikungunya and Zika [14], and yellow fever [15]. Consequently, health security has become increasingly important within the broader context of health systems-strengthening, enhancing responses to public health emergencies, and global catastrophic biological risks [16, 17].

The prioritization of GHS interventions is done both at national public health systems and in coordination with multilateral institutions including WHO, Global Health Institute, and the World Bank Group, among other institutions [2, 18–22]. The global disruption of the COVID-19 pandemic has once again reminded the world of the need to conflate national health with global health risks. WHO requires member countries to improve capacity in emerging epidemic threats as part of their commitment to safeguarding health [23]. However, the COVID-19 pandemic exposed capacity gaps which indicate that many countries were not sufficiently ready for major health risks [24, 25]. The commitment to safeguarding health often needs the measurement of GHS to evaluate countries' capacities [17]. Despite the serious threats posed by pandemics globally, compliance to commitments to health protection remains low [26].

While the GHS index provides for a framework to assess the capacity of countries to prevent and mitigate emerging health risk, the extent of the variation and the levels of contributions of a country’s health system building blocks and socioeconomic indicators to level of preparedness to epidemics and pandemics is not well known. Additionally, there is absence of evidence on whether the effects of the health system essentials differ for countries with weak and strong levels of preparedness. Studies underscore the importance of these health systems building blocks, especially in acting as a first line of defense and signaling resilience during outbreaks of health risks [27–30]. On the other hand, evidence shows how a poor health system that lacks basic inputs or building blocks cannot prepare for or respond to crises such as pandemics, posing a threat to health security [27, 31–33]. The absence of a robust health system has been shown to impede effective response during health crises across high and low income countries [27]. The main goals of this paper were to provide empirical evidence in filling this research gap on whether the effects of health systems building blocks and socioeconomic indicators differ across the distribution of countries’ levels of preparedness to health risks and examine WHO regional differentials during Covid-19 and pre-pandemic period.

First, using quantile regression, we examine the role of health system building blocks and show their differential effects along the distribution of the levels of preparedness to health risks. The GHS index was generated from 37 indicators and 96 sub-indicators [1]. Second, using logarithmic transformed data, we examined the respective share of contributions of the health system building blocks and socioeconomic factors to the overall global health security score. We interpret these share contributions as percentage changes or elasticities, establishing whether protecting GHS is a normal and necessary endeavor, consistent with public health as a collective benefit. Third, we assessed the regional differential effects of GHS during the Covid-19 and pre-pandemic periods using a regression model with time-fixed effects accounting for unobserved heterogeneity.

The rest of the study is organized as follows: Section 2 presents the methods including conceptual framework, specifications, and data. Section 3 reports results. Section 4 discusses results. Section 5 concludes.

## Methods

### Conceptual framework

The framework for analyzing health systems building blocks, socioeconomics, geography, and preparedness to health risks comes from the following generic aggregate function:

1 $$G=f\ \left(P,D,R\right)$$

where G is a measure of GHS index as a function of a vector of health system building blocks, including improved health service delivery e.g., beds per 1000 population, the health workforce development e.g. doctors and nurses per 1000 population, information systems e.g., communications infrastructure, access to essential medicines for supply chain in medicine distribution, health system financing and leadership and governance effectiveness: D is socioeconomic and development measures including SDGs, HDI, and level of income categories per World Bank classification; R represents the WHO regional offices.

### Estimable models

To examine the differential effects of global health systems building blocks and socioeconomic factors along the distribution of levels of preparedness to health risks, we specified a quantile regression model equation. We also specified a log-linear and double logarithmic models. Quantile regression differentially weights the distances between the values predicted by the regression line and the observed values, then tries to minimize the weighted distances [34]. The method has the advantage in that it allows for understanding relationships between variables outside of the mean of the data. Quantile regression weights different portions of the sample to generate coefficient estimates, thus increasing the power to detect differences in the upper and lower tails. This approach has previously been used in health services and health economics studies [34–38]. We report median regression given that it is more robust to outliers than least squares regression. The quantile regression model equation for the 𝜏^th^ quantile as:

2 $$Q\left({g}_i\right)={\beta}_0\left(\tau \right)+{\beta}_1\ \left(\tau \right){x}_{i1}+\dots \dots \dots \dots \dots +{\beta}_1\left(\tau \right){x}_{ip}\ I=1,\dots ..,n$$

where *g*_*i*_ is a measure of GHS index and *x*_*i*_ is a vector of explanatory variables, including pillars of health systems building blocks, socioeconomic and development measures, and geography. The coefficients, *β*, are functions of the quantiles, *τ* and are determined by minimizing the median absolute deviation.

3 $$MAD=\frac{1}{p}{\sum}_{n=1}^p{p}_{\tau}\left({g}_i-\Big({\beta}_0\left(\tau \right)+{\beta}_1{x}_{i1}\left(\tau \right)+\dots +{\beta}_p{x}_{ip}\left(\tau \right)\right)$$

We specify and estimate the following explicit baseline linear statistical equations. We ran both ordinary least square (OLS) and quantile regression for this specification as a comparison to illustrate how conclusions can differ when understanding effects across the entire distribution of the GHS index.

4 $${GHS}_j={\beta}_0+{\beta}_1{D}_j+{\beta}_2{N}_j+{\beta}_3{B}_j+{\beta}_4{S}_j+{\beta}_5{G}_j+{\beta}_6{F}_j+{\beta}_7{C}_i+{\beta}_8{SDG}_j+{\beta}_9{HDI}_j+{\beta}_{10}{Y}_j+\boldsymbol{\upphi} year2021+{\varepsilon}_j$$

where GHS is the overall global health security of country j; D is doctors per 1000 persons, N is nurses per 1000 persons, B is beds per 1000 persons, S is supply chain capacity, G is governance effectiveness, F is public health financing, C is communications infrastructure, SDG is social development goals, HDI is human development index, Y is World Bank’s development income level, implying Y = 1 for high income and Y = 0 for low income; Year is a dummy taking one for the Covid-19 pandemic year 2021 and zero for 2019.

Next, to estimate the share of contributions of pillars of health security to the overall GHS score, we implemented a double logarithmic regression model. Additionally, we used a log-linear model as a robustness check and to normalize the skewed distribution of the global health security index. The fitted estimable double logarithmic model is as follows:

5 $$Ln{GHS}_j={\beta}_0+{\beta}_1\ln \left({D}_j\right)+{\beta}_2\ln \left({N}_j\right)+{\beta}_3\ln \left({B}_j\right)+{\beta}_4\ln \left({S}_j\right)+{\beta}_5\ln \left({G}_j\right)+{\beta}_6\ln \left({F}_j\right)+{\beta}_7\ln \left({C}_j\right)+{\beta}_8\ln \left({SDG}_j\right)+{\beta}_9\ln \left({HDI}_j\right)+\boldsymbol{\alpha} {\sum}_{\boldsymbol{j}=1}^{4-1}{Y}_{ij}+\boldsymbol{\upphi} year2021+\boldsymbol{\lambda} {\sum}_{j=1}^{6-1}{Region}_{ij}+{\varepsilon}_j$$

where the variables are as defined earlier; Y_ij_ are income categories – low, lower middle, upper middle, and high; and regions are the WHO region groups.

Next, we implemented a regression model with time-fixed effects. The effects of the health systems building blocks and socioeconomic indicators on GHS are mediated by the differences across WHO regions and the shock of the Covid-19 pandemic as proxied by year 2021, accounting for unobserved heterogeneity.

6 $$\mathit{\ln}{GHS}_j={\beta}_0+{\beta}_1\mathit{\ln}\left({D}_j\right)+{\beta}_2\mathit{\ln}\left({N}_j\right)+{\beta}_3\mathit{\ln}\left({B}_j\right)+{\beta}_4\mathit{\ln}\left({S}_j\right)+{\beta}_5\mathit{\ln}\left({G}_j\right)+{\beta}_6\mathit{\ln}\left({F}_j\right))+{\beta}_7\mathit{\ln}\left({C}_j\right)+{\beta}_8\mathit{\ln}\left({SDG}_j\right)+{\beta}_9\mathit{\ln}\left({HDI}_j\right)+{\alpha} {\sum}_{i=1}^{4-1}{Y}_{ij}+{\upphi} year2021+{\lambda}_r{\sum}_{r=1}^{6-1}{Region}_{jr}+\pi Year2021\ast {\sum}_{r=1}^{6-1}{Region}_{jr}+{\varepsilon}_j$$

where ϕ is time trend; λ denotes a vector of WHO regional differences in GHS compared to the reference region; and π denotes difference in changes over time.

### Data

The data used were publicly available [1]. The main outcome variable we analyzed is the overall 2021 GHS Index, which measures the capacities of 195 countries to prepare for epidemics and pandemics, including threats potentially more devastating than COVID-19. However, we report and analyze for both 2019 and 2021. We recognize that the GHS index for each country is assumed to be correlated over time such that the two periods can control for unobserved characteristics that do not change or change slowly over time. Of interest were also several explanatory variables, including health systems building blocks such as human and capital resources, supply chain, public health spending, effective governance, and communication infrastructure. We draw these data from the GHS index report and the global health observatory of the WHO [39]. Other independent variables considered were socioeconomic and development measures including SDGs, HDI, and level of income category per World Bank classification as well as WHO region classification based on geography. We filtered countries by region and income level.

All variables were normalized to a scale of 0 to 100. Other data of interest include time trend from 2019 to 2021 and WHO regional offices.

We analyzed the data using STATA® version 16 (STATA Cooperation, TX). We present descriptive and regression results. We grouped countries into WHO regions and the World Bank income categories. 

## Results

This section presents the results of the descriptive analysis and various regression models for 195 countries for the data of the years 2019 and 2021 data (*N* = 390).

### Descriptive analysis

**Fig. 1:**
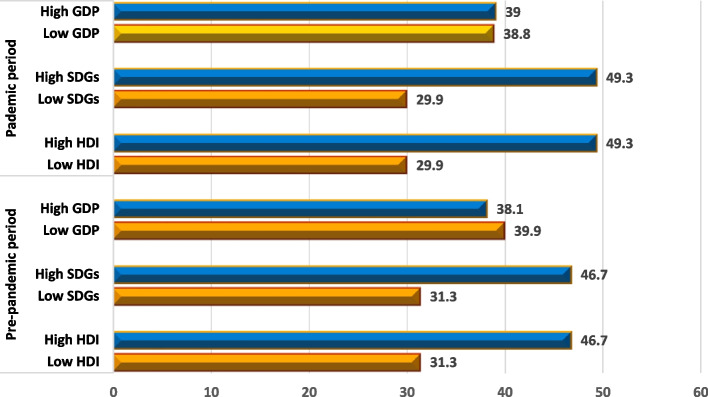
Level of preparedness to health risk by socioeconomic indicators (Mean, 0 to 100), *N*=390: Compared to pre-pandemic period, countries with low SDGs, HDI, and income were less prepared for health risks during Covid-19 pandemic

Compared to pre-pandemic period, countries with low SDGs, HDI, and income were less prepared for health risks during Covid-19 pandemic (Fig. 1). In the next section, we assess whether these effects varied with quantiles of health security.

### Quantile regression model results

Table 1 depicts the results of a level-level OLS and quantile regression analyses. The results suggest that health system building blocks such as supply chains, public health spending, and effective governance were associated with levels of preparedness to health risks. A unit increase in the score of each of these health systems building blocks was associated with an increase of between 0.08 and 0.27 in the GHS index. Increases in effective governance, supply chain capacity in terms of medicines and technologies, and financing had positive effects on the level of preparedness to health risks. These effects differ considerably, having a strong impact at higher quantiles. However, while an increase in communications infrastructure had positive effects on the level of preparedness to health risks at the 25th and 50th quantiles, a negative effect was associated at higher quantiles of preparedness to health risks.

**Table 1:** Level OLS and quantile regressions on global health security index, *N* = 390

		Quantile regression (GHS index)				
VARIABLES	OLS	10th Percentile	25th Percentile	50th Percentile	75th Percentile	90th Percentile
Year (Pre-pandemic, 2019 = reference)						
Pandemic (2021)	−2.910***	−3.633***	− 3.557***	− 2.596***	−1.953**	− 1.715*
	(0.734)	(1.383)	(0.945)	(0.801)	(0.884)	(0.995)
Doctors per 1000 persons	0.00562	−0.0189	0.0213	0.0527	0.0401	− 0.0244
	(0.0325)	(0.0825)	(0.0404)	(0.0655)	(0.0424)	(0.0427)
Nurses per 1000 persons	0.0248	0.0102	0.00879	0.0439	0.0514	−0.0109
	(0.0357)	(0.113)	(0.0398)	(0.0522)	(0.0583)	(0.0393)
Beds per 1000 persons	−0.0342	−0.0119	− 0.000324	−0.0734*	− 0.0774	0.0114
	(0.0327)	(0.121)	(0.0396)	(0.0435)	(0.0530)	(0.0409)
Supply chain capacity	0.231***	0.170***	0.230***	0.193***	0.268***	0.275***
	(0.0203)	(0.0332)	(0.0265)	(0.0262)	(0.0360)	(0.0279)
Govt health spending % public spending	0.0900***	0.0932	0.105***	0.109**	0.109***	0.0848***
	(0.0294)	(0.0829)	(0.0353)	(0.0425)	(0.0348)	(0.0251)
Governance effectiveness	0.138***	0.142***	0.1000***	0.116***	0.133***	0.214***
	(0.0281)	(0.0452)	(0.0345)	(0.0335)	(0.0416)	(0.0377)
Communication infrastructure capacity	0.0549*	0.0576	0.0942**	0.0719**	−0.0135	−0.000723
	(0.3090)	(0.0649)	(0.0419)	(0.0351)	(0.0550)	(0.0355)
Human development index (HDI)	4.821**	2.740	3.137	6.715**	5.849	3.783
	(2.068)	(4.462)	(2.454)	(2.709)	(4.355)	(4.261)
Social development goals (SDGs)	0.147***	0.119***	0.111***	0.127***	0.143***	0.166***
	(0.0190)	(0.0415)	(0.0224)	(0.0209)	(0.0229)	(0.0229)
World Bank Development level (Low-income = reference)						
High-income	−0.512	−1.808	−2.286*	−2.273*	0.759	1.868
	(1.020)	(1.490)	(1.224)	(1.259)	(1.395)	(1.940)
Constant	11.52***	9.306***	10.14***	11.92***	17.18***	17.41***
	(1.600)	(3.109)	(1.997)	(2.168)	(2.924)	(1.620)
Observations	390	390	390	390	390	390
R-squared	0.779	0.768	0.771	0.772	0.772	0.769

Standard errors in parentheses; *** *p* < 0.01, ** *p* < 0.05, * *p* < 0.1

**Fig. 2:**
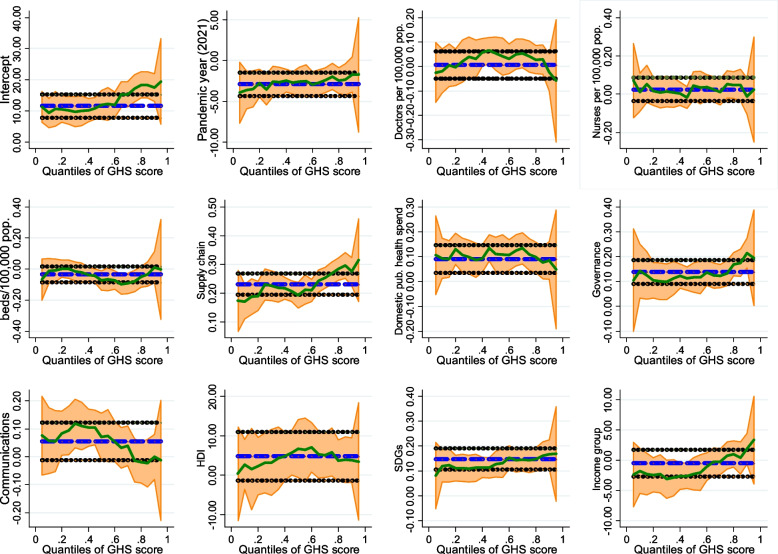
indicates comparison of the different effects of the factors controlled for in the OLS and quantile regressions

A unit score increases in the SDG score had a positive effect on the level of preparedness to health risks. This effect differs considerably, having a strong impact on the GHS index at higher quantiles (*p* < 0.001). Compared with countries with lower income, countries with higher income had 2.2 lower GHS scores at the 25th and 50th quantile (*p* < 0.001). This differential effect was not significant at higher quantiles. While positive effects of SDGs are greater for countries with higher levels of preparedness to health risks, the effect of HDI on the level of preparedness is greatest for countries whose overall GHS index lies at midpoint of the frequency distribution of observed values Fig. 2.

### Log-linear and double-logarithmic models results

Table 2 shows the results of log-linear and double-logarithmic regressions. According to the log-linear regression, a unit increase in each of the health system building blocks such as supply chain, public health spending, effective governance, and communication infrastructure were associated with a 0.59, 0.18, 0.41, and 0.41% increase in the geometric mean of the GHS index. A unit increase in the SDG score was associated with a 0.37% increase in the geometric mean of the GHS index. A unit increase in the HDI score was associated with a 24% increase in the GHS index. However, the pandemic year (2021) was associated with a 7.4% decrease in the geometric mean of the GHS index (*p* < 0.001). There is a negative gradient between level of income and the level of preparedness to health risks. Relative to poor countries, middle- and high-income countries had lower levels of preparedness to health risks, an indication of a sense of complacency.

**Table 2:** Log-linear and Log-log regression on global health security index, *N* = 390

	Log-linear	Log-log	
Year (reference = Pre-pandemic, 2019)		Year (reference = Pre-pandemic, 2019)	
Pandemic (2021)	− 0.0767***	Pandemic (2021)	− 0.0696***
	(0.018)		(0.019)
Doctors per 1000 persons	0.001	Doctors per 1000 persons ^a^	−0.005
	(0.001)		(0.005)
Nurses per 1000 persons	0.000	Nurses per 1000 persons ^a^	0.0161***
	(0.001)		(0.004)
Beds per 1000 persons	−0.001	Beds per 1000 persons ^a^	0.002
	(0.001)		(0.005)
Supply chain capacity	0.00586***	Supply chain capacity ^a^	0.0125***
	(0.001)		(0.001)
Govt health exp. % public exp.	0.00182**	Govt health exp. % public exp. ^a^	0.0111***
	(0.001)		(0.004)
Governance effectiveness	0.00414***	Governance effectiveness ^a^	0.144***
	(0.001)		(0.021)
Communication infrastructure	0.00415***	Communication infrastructure ^a^	0.011
	(0.001)		(0.011)
HDI	0.215***	HDI ^a^	0.008
	(0.060)		(0.005)
SDGs	0.00372***	SDGs ^a^	0.0155***
	(0.001)		(0.002)
World Bank income category (Low income = reference)			
Low-middle income	−0.0647*		−0.0768**
	(0.033)		(0.036)
High-middle income	−0.108**		−0.038
	(0.042)		(0.047)
High-income	−0.204***		0.022
	(0.051)		(0.053)
WHO regions (Africa = reference)			
Americas	0.017		0.135***
	(0.036)		(0.041)
Eastern Mediterranean	−0.029		0.053
	(0.036)		(0.037)
European	−0.012		0.225***
	(0.042)		(0.039)
Southeast Asia	0.126***		0.205***
	(0.044)		(0.055)
West Pacific	−0.030		0.061
	(0.036)		(0.045)
Constant	2.743***		2.936***
	(0.045)		(0.082)
Observations (n)	390		390
R-squared	0.798		0.731

Robust standard errors in parentheses; *** *p* < 0.01, ** *p* < 0.05, * *p* < 0.1

^a^ implies variable in natural logarithm

In the log-log model, the marginal contributions to the level of preparedness to health risks of human resources, supply chains, public health financing and governance of the healthcare systems were significant. A 10% increase in each of the health system building blocks was associated with an increase of between 0.11 to 1.4% in the GHS index (all *p* < 0.001). Thus, as expected, the stronger the health system building blocks, the stronger the preparedness of countries to threats of epidemics and pandemics. A 10% increase in the SDG index was associated with a 0.15% increase in the GHS index (< 0.001). The year 2021 (during the pandemic) was associated with a lower GHS index compared to the pre-pandemic period. Compared to African region, the Americas, European, and Southeast Asia regions were associated with higher GHS. There was statistically significant difference between African region and the rest of the WHO regions.

### A regression model with time fixed effects

**Fig. 3:**
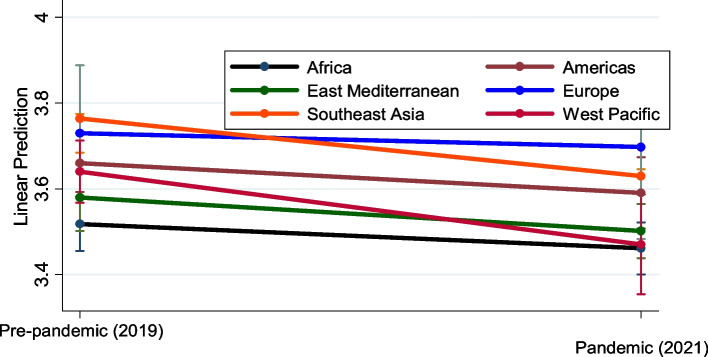
Predictive margins of trends in preparedness to health risks and WHO regional differentials: A negative trend in the level of preparedness to health risks from 2019 to 2021 for all regions is shown, implying all regions were less prepared during Covid-19 pandemic compared to pre-pandemic period

The results of the effects of health systems building blocks and socioeconomic indicators in the time fixed-effects model corroborate with the results of the previous specifications. The results in Table 3 show that there were significant regional differences in GHS. Specifically, GHS was higher on average in the Southeast Asia, Europe, Americas, and West Pacific regions than in Africa. Although not statistically significant, the parameter estimate of the Eastern Mediterranean region implies that it also had higher GHS. This would suggest that the African region was the least prepared to health risks across all the regions. These results corroborate with the depiction of Fig. 3.

**Table 3:** A regression model with time fixed effects, *N* = 390

VARIABLES	Dependent variable = Natural logarithm of GHS index	Robust S.E
Doctors per 1000 persons	−0.00540	(0.00522)
Nurses per 1000 persons	0.0163***	(0.00400)
Beds per 1000 persons	0.00190	(0.00533)
Supply chain capacity	0.0124***	(0.00142)
Govt health spending % public spending	0.0109**	(0.00442)
Governance effectiveness	0.142***	(0.0206)
Communication infrastructure capacity	0.0101	(0.0106)
Human development index (HDI)	0.00871	(0.00548)
Social development goals (SDGs)	0.0168***	(0.00241
World Bank income category (low-income = reference)		
Low-middle income	−0.0763**	(0.0367)
High-middle income	−0.0357	(0.0469)
High-income	0.0264	(0.0528)
Year (2021)	−0.0571*	(0.0330)
WHO regions (Africa = reference)		
Americas	0.142***	(0.0500)
Eastern Mediterranean	0.0613	(0.0519)
European	0.211***	(0.0452)
Southeast Asia	0.246***	(0.0694)
West Pacific	0.122**	(0.0498)
Year * Americas	−0.0126	(0.0613)
Year * Eastern Mediterranean	−0.0208	(0.0612)
Year * European	0.0255	(0.0447)
Year * Southeast Asia	− 0.0776	(0.0975)
Year * West Pacific	−0.113	(0.0774)
Constant	2.931***	(0.0840)
Observations	390	
R-squared	0.735	

Robust standard errors in parentheses

*** *p* < 0.01, ** *p* < 0.05, * *p* < 0.1

## Discussion

This study aimed to examine the differential effects of health systems building blocks and socioeconomic factors along the distribution of countries’ level of preparedness to health risks. The study also examined WHO regional offices’ preparedness level differentials during Covid-19 and pre-pandemic period.

The quantile regression results suggest that the effect of effective governance, supply chain capacity in terms of medicines and technologies, and financing had positive effects on the level of preparedness to health risks, with impact being considerably larger for countries with higher levels of preparedness to health risks. These positive gradient trends signal a sense of capacity on the part of countries with higher global health security.

More generally, evidence shows that a well-functioning health system act as a first line of defense during outbreaks of health risks [27]. With increasingly global health risks, there is a need to have well-integrated and locally grounded health systems that are more resilient to shocks. Such a need should include designing efficient health information systems, financing mechanisms, and health workforces. These building blocks imply having the information and knowledge to make a decision on what needs to be done, and investing or mobilizing resources to fund a response [28]. To ensure interventions in all health system building blocks are successful, such designs should also recognize and include promoting effective governance and wider systems values [28]. Governance challenges in health systems were remarkably noticeable during Covid-19 pandemic, including weak organizational coordination, inefficient inter-sectoral relationships, parallel decisions, inefficient distribution of the human resources, lack of applied education, lack of integrated health protocols, and lack of appropriate evaluation of performance [28].

Our results suggest that health-related workforces as building blocks of a well-functioning health system were not associated with commensurate levels of health risk preparedness. While an increase in nurses per 1000 population was the only factor in this category to be associated with increased preparedness to health risk, even then, we find no statistically significant difference between low and high prepared countries. The health workforce is crucial for a health system because it is the component that determines how plans for response to shocks are implemented [28]. However, our results indicate that although an increase in doctors per 1000 population enhanced level of preparedness to health risks, that impact was not statistically significant. Frontline workers in communities were found to be important assets in the capacity building and preparedness strategies during Covid-19 pandemic [29]. Health systems faced several health workforce challenges, including inefficient distribution, employee turnover, lack of clear approaches for staffing, and shortage of specialized manpower [30]. Many personnel-related challenges were noted, including insufficient knowledge of the employees, psychological disorders, reduction of self-confidence, burnout, workload increase, reduced level of job satisfaction, effects of colleague and patients bereavement and unsafety sense against the work place [30].

A notable finding in our study is that compared to other building blocks, the supply chain was associated with a higher impact on the level of preparedness to health risks, with the impact being considerably larger for countries with higher quantiles of preparedness. Increase in supply chains, notably medicines and technologies appear to have enhanced the level of preparedness to health risks, with impact being considerably higher in more prepared countries. It has been noted that the ability for rapid development of medical products and being able to take procurement and manufacture of new products to scale in a very short time period was a key resilience and health systems preparedness lesson during Covid-19 pandemic [40]. However, our results suggest that while an increase in communications infrastructure had positive effects on the level of preparedness to health risks at lower quantiles, a negative effect was associated at higher quantiles of preparedness to health risks. The impact was considerably greater for countries with lower levels of preparedness to health risks. Generally, while the emphasis of a robust health system is on good disease surveillance systems and their integration with health management information systems [40], it appears this was impactful in less prepared countries than in more prepared economies. Communications infrastructures were much needed in weak health systems, given that the dearth of well-coordinated communication channels can bode ill for the successful fight against pandemics [41]. Evidence shows a lack of communication could jeopardize effective interventions to mitigate exposure and management of health risks, especially in weak health systems [42].

Further, our analysis suggests positive gradient effects of SDGs on GHS that are smaller when countries have lower global health security, but much larger when global health security is higher. Compared to pre-pandemic period, countries with low SDG index were underprepared for health risks during Covid-19 pandemic. These results affirm the interconnectedness of protection of population health and SDGs. In the world’s agenda for SDGs by 2030, ensuring good health and wellbeing remains a central goal [5, 43], and that improving public health is a central pillar for the SDGs agenda [44]. A subpoint of the SDG health goal is the strengthening of early warning, risk reduction, and management of health risks [5]. Almost 16 of the SDGs goals are related to health or their achievement will contribute to health indirectly [43] and should be a priority in global health policy dialog [45–47]. The contribution of HDI to GHS index. HDI has the greatest positive effect on countries whose overall GHS index lies at the midpoint of a frequency distribution of observed values. Compared to pre-pandemic period, countries with low HDI were underprepared for health risks during Covid-19 pandemic. This result underscores the importance of HDI to countries with median GHS index capabilities. Improving HDI provides information on the development of countries, considering essential issues that influence people’s well-being [48]. In showing the value proposition of the GHS Index, HDI has been shown as a valuable tool for guiding decision making and monitoring policymaking at both national and subnational levels, especially on health security capacities and capabilities in many countries [6].

Moreover, the results showed a negative income differential effect on the GHS index, with high-income countries being associated with lower levels of preparedness to health risks than low-income countries. However, countries with less capacity to respond to health threats generally tend to be low-income. This result is intuitive given that any increase in income in these countries is likely to be used to improve food and nutrition. Improvement in food and nutrition can in turn enhance health. Evidence shows that food and health security are attainable only when the underlying social inequities are addressed [49]. Improvements in incomes in developing countries do not necessarily translate to enhancements in GHS in the short run because these countries face challenges of food security, nutrition, and poverty.

Interestingly, while quantile regression reveals negative and positive gradient differential impact of health systems building blocks and socioeconomic indicators, these impacts are masked the OLS estimation. The logarithmic regression showed the marginal contributions to the level of preparedness to health risks of nurses per 1000 population, supply chain, public health financing and governance of the healthcare systems were positive and significant. The percentage changes in the GHS index with respect to percentage changes in the health systems building blocks imply that preparedness to health risks is a normal and necessary endeavor. Given the increasing regularity with which infectious disease threats happen, the results underscore the fact that it is imperative upon countries across the globe to enhance GHS preparedness regardless of the changes in health systems building blocks. The idea that promotion of GHS is a necessary endeavor is also consistent with healthcare as a necessity, especially when delivered through the public sector [50, 51]. Enhancing GHS as a collective benefit is also consistent with the good health and safe food imperatives argument. If the health and economic burden of a local issue such as unsafe food can be avoided through preventive measures, investments, and behavioral changes adopted from farm to fork, [52] countries should take a global matter as important as GHS more seriously than the current complacency and cavalier attitude. The 2021 GHS Index report showed that countries are continuing to neglect the preparedness needs of vulnerable populations, which exacerbates the impact of health security emergencies [1]. Thus, as a necessary endeavor and imperative, the promotion of GHS requires local, national, regional, and global responses to establish how an outbreak becomes a pandemic and to prepare for future health threats [53]. Thus, it is imperative to build accountability for national preparedness and in coordination with multilateral institutions including WHO, Global Health Institute, the National Academy of Medicine, and the World Bank Group [2, 18–22].

The foregoing results indicate the importance of the contributions of health systems building blocks, affirming their value proposition in enhancing health security capacity. The results further indicated that the year 2021 (during the pandemic) was associated with a statistically significant less preparedness to global health risks compared to the pre-pandemic period. Again, this result affirms that, as has been shown during Covid-19 outbreak, the health security of countries remains fragile and that no countries were sufficiently ready for a major biological [24, 25, 54].

The results showed statistically significant regional differences in the level of preparedness to health risks. Compared to African region, the Americas, European, and Southeast Asia regions were associated with higher GHS. There was statistically significant difference between African region and the rest of the WHO regions.

The results of the time-fixed effect comparison of the changes in GHS from 2019 to 2021 show the positive effects of health systems building blocks and socioeconomic indicators. Apart from the Eastern Mediterranean region, the rest of the WHO regions were more prepared to health risks compared to Africa. There was a negative time trend in the level of preparedness to health risks, although regional differences in changes over time were not statistically significant. The 2021 GHS index report showed that most countries saw little or no improvement in maintaining a robust, capable, and accessible health system for outbreak detection and response [1]. The suggestion that some regions performed better than other regions is consistent with WHO data during the Covid-19 pandemic. For instance, the Western Pacific region recorded the highest total vaccine doses administered of 222.52 per 100 population, while European region registered 166.89 per 100 population. Western Pacific countries also recorded the highest in terms of persons boosted, registering 46.78 per 100 population. European region was the third after Western Pacific and the Americas, registering 27.55 per 100 population. The two regions were way above the global average, both in terms of total vaccine doses administered and persons boosted per 100 population [55]. There is evidence that countries in Asia-Pacific region such as Taiwan and New Zealand had global successes in strategies to control COVID-19 compared to countries in Western Europe. Countries in this region took urgent action to eliminate community transmission through a series of non-pharmaceutical interventions: a ‘zero-COVID’ strategy. At the same time, they kept their economies afloat and avoided longer, harsher lockdown measures [56].

### Contribution and limitations

This study is the first to examine whether the effects or contributions and strength of the health systems building blocks and socioeconomic indicators on countries’ level of preparedness to health risks differ for countries with weak and strong health securities. At the same time, the study assessed the regional differential effect of GHS during Covid-19 and pre-pandemic period However, the study has limitations. It relied on macro data from the 2021 global health security index. The index has been criticized for showing a discrepancy between the GHS index rating and the actual performance of countries during pandemic, overestimating the preparedness of some and underestimating others [57]. A more microdata disaggregating the preventive and responsive measure of countries as well as robustness of health systems, commitments and overall risks would have provided a more accurate behaviors of individuals in communities in different countries in preparing to and response to global health risks. Broadly, microdata can be beneficial in exploring the rich sources of heterogeneity shaping the behaviors of participants at the micro level of society. Microdata also help in netting out a large array of individual-level factors that may contribute to geographic variation in health care utilization [58]. The use of more microdata can improve on aggregate time-series methods by building models that link economic models for individuals to data on individual behavior [59].

## Concluding remarks

In conclusion, we revisit my original queries. What is the impact of health systems building blocks and socioeconomic indicators on level of preparedness to epidemics and pandemics, and whether such effects differ for less and more prepared countries?

Our analysis of the relationship between health systems building blocks, socioeconomics, and regional differences versus preparedness to health risks consistently confirmed the robustness of the models estimated. The choice of the specifications also corroborates with model statistics and internal validity assessments, including mis-specification test tool (Linktest) available in Stata, the econometric software used in the research. The direction and magnitude of the coefficients reveal the contribution of each of the health systems building blocks, socioeconomic indicators, and regional differentials to the overall level of preparedness to health risks. The results show that increases in effective governance, supply chain capacity in terms of medicines and technologies, and financing had positive effects on the level of preparedness to health risks. However, the health workforce including doctors, and health services including hospital beds were not statistically significant in explaining the variations in countries’ level of preparedness.

Using a quantile regression, we show that the effect of effective governance, supply chain capacity in terms of medicines and technologies, and financing had positive effects on the level of preparedness to health risks, with impact being considerably larger for countries with higher levels of preparedness to health risks. These positive gradient trends signal a sense of capacity on the part of countries with higher global health security. While socioeconomic factors had positive effects on the level of preparedness to health risks, their impacts on the distribution of countries’ level of preparedness to health risks were mixed. The effects of SDGs were greater for countries with higher levels of preparedness to health risks. The effect of HDI on the level of preparedness was greatest for countries whose overall GHS index lie at the midpoint of the distribution of the level of prepareness. High-income was associated with a negative effect on the level of preparedness, especially if countries were in lower quantiles across the distributions of preparedness. Relative to poor countries, middle- and high-income countries had lower levels of preparedness to health risks, an indication of a sense of complacency.

All the models reveal that the pandemic period (year 2021) was associated with a decrease in the level of preparedness to health risks compared to the pre-pandemic period. There were significant regional differences, and apart from the Eastern Mediterranean region, the rest of the WHO regions were more prepared to health risks compared to Africa. There was a negative time trend in the level of preparedness to health risks from 2019 to 2021. However, regional differences in changes over time were not statistically significant.

We conclude with implications and recommendations for practical actions for addressing health systems building blocks and socioeconomic indicators impacting on GHS preparedness. Our results would suggest that attempts to strengthen countries’ level of preparedness to health shocks should be focused more on enhancing essentials such as supply chain capacity in terms of medicines and technologies; health financing, communication infrastructure, while maintaining their already existing health workforce and health services. Strengthening health systems building blocks would be beneficial to less prepared countries where their impacts we find to be weaker. Similarly, boosting SDG, particularly health-related sub-scales, would be beneficial to less prepared countries. There is a need to curb complacency in preparedness to health risks during pandemics by high income and countries with better capacity for protecting population health. The negative trend in the level of preparedness to health risks would suggest that there is a need for better preparedness during pandemics by conflating national health with global health risks. This will ensure the imperative of having synergistic response is apparent to all countries and regions.

## Data Availability

Data supporting study findings are available upon request.

## Abbreviations

AFRO: African regional Office
AMRO: Americas regional office
CDC: The Center for Disease Control
EMRO: Eastern Mediterranean regional office
EURO: European regional office
GDP: Gross domestic product
GHS: Global health security
HDI: Human development index
IHR: The International Health Regulations
MERSCoV: Middle East respiratory syndrome coronavirus
OLS: Ordinary least square
SARS: Severe acute respiratory syndrome
SEARO: Southeast Asia regional office
SDGs: Sustainable development goals
WHO: The World Health Organization
WPRO: West Pacific regional office
